# Limbus- Versus Fornix-Based Trabeculectomy for Open-Angle Glaucoma Eyes with Prior Ocular Surgery: The Collaborative Bleb-Related Infection Incidence and Treatment Study

**DOI:** 10.1038/srep09290

**Published:** 2015-03-19

**Authors:** Satoshi Yokota, Yuji Takihara, Masaru Inatani

**Affiliations:** 1 Department of Ophthalmology, Faculty of Medical Science, University of Fukui, Fukui, Japan; 2 Department of Ophthalmology and Visual Sciences, Kyoto University Graduate School of Medicine, Kyoto, Japan

## Abstract

We compared the surgical successes of limbus- and fornix-based trabeculectomies in open-angle glaucoma (OAG) eyes that had prior ocular surgery in the Collaborative Bleb-Related Infection Incidence and Treatment Study (CBIITS), Japan. From the 1,098 glaucoma eyes in 34 clinical centers in CBIITS, 195 OAG eyes that had undergone previous trabeculectomy and/or lens extraction were included. Limbus- or fornix-based trabeculectomy with mitomycin C were performed. Surgical failure (IOP ≥ 21, 18, or 15 mmHg for criterion A, B or C, respectively; <20% decrease from baseline; reoperation for glaucoma; or loss of light perception vision) was counted. There were 106 and 89 eyes treated with limbus- and fornix-based trabeculectomies, respectively. At 3 years, IOP (mean ± SD) was 12.5 ± 5.9 and 14.1 ± 6.4 mmHg and the cumulative probabilities of failure during 3 years were 30.2% and 50.5% for criterion A, 40.3% and 57.4% for criterion B, and 57.9% and 65.8% for criterion C in the limbus- and the fornix-based group, respectively. Fornix-based incisions were associated with surgical failure in Cox-proportional multivariable analysis for criterion A [relative risk (RR) = 1.96], and B [RR = 1.60]. Limbus-based trabeculectomy had a higher probability of success in OAG eyes with prior ocular surgery.

Trabeculectomy is the gold standard surgery for lowering intraocular pressure (IOP) in glaucoma patients^1^. Because the IOP-lowering effect depends on the formation of the filtering bleb in the conjunctiva, surgical success is affected by the subconjunctival fibrosis present before, during, and after trabeculectomy. Many risk factors for trabeculectomy have been previously described. Non-Caucasians and younger patients show more resistance to trabeculectomy because fibroblasts in the subconjunctiva are suggested to be more active^2^. Intraocular inflammation appears to reduce the success rate because uveitic glaucoma or previous intraocular surgery are risk factors for surgical failure of trabeculectomy^3^. In addition, neovascularization has been associated with bleb failure because neovascular glaucoma is more resistant to trabeculectomy than primary open-angle glaucoma (POAG)^4^.

Lens extraction or prior trabeculectomy are also risk factors for trabeculectomy failure in open-angle glaucoma eyes^5^,^6^. Surgical scarring in the conjunctiva can be caused by a prior lens extraction or trabeculectomy. Pseudophakic eyes often exhibit elevated inflammatory cytokines in the aqueous humor^7^. These adverse factors seem to promote bleb failure after trabeculectomy. This was further supported by a tube versus trabeculectomy study that investigated the comparison of surgical outcomes between tube shunt surgery versus trabeculectomy in mainly open-angle glaucoma (OAG) eyes that had a prior trabeculectomy or lens extraction^8^,^9^. This study revealed that trabeculectomy resulted in more surgical failures in these cases than tube shunt surgery. However, 53.1% of the trabeculectomy group showed surgical success at 5 years^9^, which suggested that these cases may have been at a high risk for surgical failure, but were not completely resistant to trabeculectomy. The choice of a conjunctival flap incision during trabeculectomy may attenuate bleb failure in these cases^10^.

There have been several indications that the creation of the conjunctival flap during trabeculectomy can affect the morphology of the filtering bleb^11^,^12^. Fornix-based conjunctival flap incisions provide a more vascular, diffuse, and extended bleb than limbus-based conjunctival flap incisions. Because the pre-existing conjunctival scarring prevents bleb expansion, a fornix-based trabeculectomy may not allow the development of diffuse or extended filtering blebs in these cases. By contrast, avascular and cystic blebs created by limbus-based trabeculectomy procedures may contribute to the surgical outcome in these cases. Although there are many retrospective and prospective studies about the comparison of surgical outcomes between limbus- versus fornix-based trabeculectomy, most of the studies have not shown significant better surgical outcomes with limbus-based trabeculectomy than fornix-based trabeculectomy^13^,^14^,^15^,^16^,^17^,^18^,^19^,^20^,^21^,^22^,^23^,^24^. This may be due to the small sample size or the use of patients with various backgrounds that comprised low and high risk factors. One retrospective study showed a significantly better prognosis with limbus-based trabeculectomy than with fornix-based trabeculectomy for pseudophakic OAG eyes^10^.

The Collaborative Bleb-Related Infection Incidence and Treatment Study (CBIITS) is a prospective multicenter study that investigated the incidence, severity, and prognosis of bleb-related infections after trabeculectomy with mitomycin C (MMC)^25^. This study also included 1,098 cases with either limbus- or fornix-based trabeculectomy, which represented various risk factors for surgical failure. IOPs and bleb vascularity were also recorded during the follow-up period. The aim of this study was to determine whether a limbus-based conjunctival incision contributed to better surgical outcomes after trabeculectomy for OAG eyes that had a previous trabeculectomy or lens extraction by analyzing a large sample of eyes in the CBIITS.

## Methods

### Patient Selection

The detailed protocol of CBIITS has been reported elsewhere^26^. In brief, the enrollment period was 2 years and follow-up was conducted every 6 months for up to 5 years. Ophthalmological examinations were completed at each follow-up visit according to the protocol. Thirty-four institutions participated in CBIITS. The experimental protocols were approved by the Gifu University Graduate School of Medicine. Written informed consent was obtained from all patients after a detailed explanation of the procedures involved. The study was performed in accordance with approved guidelines. As previously reported^26^, CBIITS had enrolled 1,249 eyes of 1,249 cases during 2 years from all institutions. Enrollment ended on March 31, 2007. The study included 1,098 eyes that had been treated with trabeculectomy with mitomycin C. A total of 151 eyes were excluded from the 1,249 subjects in the present study for the following reasons: 38 eyes had been treated with filtering surgery other than trabeculectomy, 65 eyes had undergone trabeculectomy without MMC, and 48 cases were lost to follow-up before the one-year follow-up.

Among the remaining patients, the patients who met the following criteria were analyzed.

Inclusion criteria were:

1. Previous trabeculectomy or previous cataract extraction, or both, or
2. POAG or exfoliation glaucoma

Exclusion criteria were:

1. Previous vitreo-retinal surgery including vitrectomy and buckling surgery,
2. Previous canal surgery for glaucoma, or
3. Previous tube shunt surgery.

Patients who met these criteria were divided into 2 groups according to the conjunctival approaches during trabeculectomy; either a limbus-based conjunctival flap (the limbus-based group) or a fornix-based conjunctival flap (the fornix-based group).

### Primary Outcome Measure

The primary outcome was surgical failure based on IOP. IOPs after trabeculectomy were recorded at each clinical center, and the mean IOPs for every 6 months after trabeculectomy were sent to the CBIITS data analysis center at Gifu University. The cumulative probabilities of surgical failure were compared in both groups. Surgical failure was defined as the following mean IOPs for every 6 month interval after trabeculectomy with or without anti-glaucoma medications: less than 20% reduction of the preoperative IOP or ≥21 mm Hg (criterion A), ≥18 mm Hg (criterion B) or ≥15 mm Hg (criterion C). However, surgical failure was declared for all criteria in cases that required reoperation for glaucoma or developed a loss of light perception vision. Reoperation for glaucoma was defined as a bleb needling more than 6 months after the trabeculectomy, bleb revision, or additional glaucoma surgery. Laser suture lysis or bleb needling within 6 months after trabeculectomy was not considered a surgical failure because it was part of the postoperative management for trabeculectomy. The incidence of needling within 6 months was not different between 2 groups (13.2% and 10.1%, limbus-based and fornix-based respectively; P = 0.505).

### Secondary Outcome Measures

Secondary outcome measures included the mean IOP postoperatively at every 6-month interval and the number of medications, the need for reoperation, bleb morphology, and postoperative complications.

### Statistical Analysis

Univariate comparisons between the 2 groups were performed using the two-sided Student t-test for continuous variables and the Chi-square test or Fisher exact test for categorical variables. The cumulative probabilities of surgical failure were analyzed using the Kaplan–Meier survival curve and log-rank test. Multivariate analysis was performed to determine the prognostic factors for surgical failure of trabeculectomy using the Cox proportional hazard regression analysis with forward stepwise elimination. A *P* value of 0.05 or less was considered statistically significant.

### Sample Size

In a previous meta-analysis report about limbus- and fornix-based trabeculectomies, IOP reduction appeared to be the same or superior in limbus-based trabeculectomy^13^. If the difference of the rates of surgical failure was 20%, the minimum sample size was calculated to be 151 eyes with a one-sided significance level of 0.05 and a power of 0.8. We speculated that the 1,098 eyes that underwent trabeculectomy with MMC in CBIITS included a sample size large enough to exhibit statistical significance.

### The follow-up period until the primary outcome measure

In CBIITS, 824 (75.0%) eyes completed the 5-year follow-up. However, in the eyes which satisfied the criteria for this study, 68.7% completed the 5-year follow-up. One reason was that the patients who previously had a trabeculectomy or lens extraction were significantly older than the patients in the total CBIITS cohort (mean age ± standard deviation, 69.4 ± 11.2 year old vs. 63.8 ± 13.0 year old, respectively; *P* < 0.0001; z-test). The primary outcome was measured at 3 years after trabeculectomy.

### Recruitment and Retention

In total, 195 patients (106 patients in the limbus-based group and 89 patients in the fornix-based group) satisfied the inclusion criteria. Twenty (18.9%) limbus-based trabeculectomy patients and 23 (25.8%) fornix-based trabeculectomy patients did not complete the 3-year follow-up visit. The number of patients who completed the 3-year follow-up visit was not significantly different between the 2 groups. (*P* = 0.243, chi-square test).

### Preoperative Characteristics

The preoperative characteristics in the limbus- and fornix-based groups are shown in Table 1. No significant differences were found between the 2 groups. Six (5.7%) eyes in the limbus-based group and 10 (11.2%) eyes in the fornix-based group had trabeculectomy combined with lens extraction (*P* = 0.158; chi-square test).

## Results

### Primary Outcome Measure

Among the patients who completed the 3-year follow-up visit, surgical failure occurred in 24 (27.9%), 32 (37.2%), and 47 (54.7%) eyes for criteria A, B, and C, respectively, in the limbus-based group. Furthermore, surgical failure occurred in 33 (50.0%), 38 (57.6%), and 44 (66.7%) eyes for criteria A, B, and C, respectively, in the fornix-based group. Significantly higher failure rates were found in the fornix-based group for criteria A (*P* = 0.005, chi-square test) and B (*P* = 0.012 chi-square test). The difference between both groups was not significant for criterion C (*P* = 0.133, chi-square test). Failure due to insufficient IOP reduction was observed in 22 (25.6%) eyes for criterion A, 30 (34.9%) eyes for criterion B, and 45 (52.3%) eyes for criterion C in the limbus-based group, whereas insufficient IOP reduction was observed in 31 (47.0%) eyes for criterion A, 37 (56.1%) eyes for criterion B, and 43 (65.2%) eyes for criterion C in the fornix-based group. Significantly higher failure rates due to insufficient IOP reduction were found in the fornix-based group for criteria A (*P* = 0.006, chi-square test) and B (*P* = 0.009, chi-square test). The difference between both groups was not significant for criterion C (*P* = 0.111, chi-square test). Surgical failure due to the loss of light perception was not observed in patients who completed the 3-year follow-up. Failure due to bleb needling after 6 months or the need for reoperation was found in 4 (4.7%) eyes of the limbus-based group versus in 4 (6.1%) eyes of the fornix-based group; this exhibited no significant difference (*P* = 0.689, chi-square test).

In the limbus-based group, success without anti-glaucoma medications (complete success) was achieved for 39 (45.3%), 37 (43.0%), and 31 (36.0%) eyes for criteria A, B, and C, respectively. In the fornix-based group, complete success was achieved for 19 (28.8%), 18 (27.3%), and 16 (24.2%) eyes for criteria A, B, and C, respectively. The rate of complete success was also significantly higher in the limbus-based group for criteria A (*P* = 0.036, chi-square test) and B (*P* = 0.044, chi-square test). No significant difference was found for criterion C (*P* = 0.116, chi-square test).

**Figure 1:**
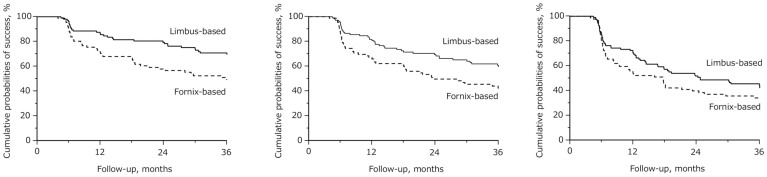
Kaplan–Meier survival curves of the probability of success in the limbus-based group and fornix-based group. (Left) For criterion A (intraocular pressure ≥ 21 mm Hg, less than 20% reduction of the preoperative intraocular pressure, reoperation for glaucoma or a loss of light perception vision), (Center) for criterion B (intraocular pressure ≥ 18 mm Hg, less than 20% reduction of the preoperative intraocular pressure, reoperation for glaucoma or a loss of light perception vision), and (Right) for criterion C (intraocular pressure ≥ 15 mm Hg, less than 20% reduction of the preoperative intraocular pressure, reoperation for glaucoma or a loss of light perception vision). A cumulative success rate was 69.8% and 49.5% (*P* = 0.003) for criterion A, 59.7% and 42.6% for criterion B (*P* = 0.012), and 42.1% and 34.2% for criterion C (*P* = 0.128) in the limbus- and fornix-based groups, respectively.

The results of the Kaplan-Meier survival curve analysis comparing the 2 groups for criteria A, B, and C are shown in the Figure 1. Among patients in the limbus-based group, surgical failure occurred in 30.2%, 40.3%, and 57.9% based on criteria A, B, and C, respectively. Among patients in the fornix-based group, surgical failure occurred in 50.5%, 57.4%, and 65.8% based on criteria A, B, and C, respectively. Significantly higher failure rates were observed in the fornix-based group for criteria A (*P* = 0.003) and B (*P* = 0.012), whereas no significant difference was found for criterion C (*P* = 0.128).

### Secondary Outcome Measures

IOPs at various follow-up time points and the numbers of anti-glaucoma eye drops were compared between the groups (Table 2). IOP levels were significantly lower in the limbus-based group compared with those in the fornix-based group at 6 months (*P* = 0.027) and 2 years (*P* = 0.027) after trabeculectomy; however, no significant differences in the number of anti-glaucoma medications between groups were found at any time points after trabeculectomy. Avascular blebs at 3 years were associated with 37 eyes (34.9%) in the limbus-based group versus 14 eyes (15.7%) in the fornix-based group, demonstrating a significant difference (*P* = 0.002; chi-square test). Despite the higher frequency of avascular blebs in the limbus-based group, there was no significant difference between the 2 groups in the frequency of bleb leaks with 5 eyes (4.7%) in the limbus-based group versus 8 eyes (9.0%) in the fornix-based group (*P* = 0.234; chi-square test). No significant differences in surgical complications were found between both groups (Table 3). The median of post operative day of re-operation was 440 and 553 (limbus-based and fornix-based, respectively). There is no significant difference between the two groups because each group has only 5 re-operation patients.

### Prognostic Factors for Failure of Trabeculectomy

Baseline characteristics, including age, sex, type of glaucoma, the number of prior trabeculectomy procedures, lens status at the time of trabeculectomy, logMAR BCVA, preoperative IOP, the number of glaucoma medications, the use of combined lens extraction, and the type of conjunctival flap incision were evaluated as possible predictors of surgical failure. In the analyses using univariate Cox proportional hazard regression models (Table 4), 3 factors were significant. Fornix-based conjunctival flaps were significant for criteria A (relative risk [RR] = 2.00; *P* = 0.004) and B (RR = 1.71; *P* = 0.013), but not for criterion C (*P* = 0.131). Lower preoperative IOP were also significant for criterion A (RR = 0.95, per mm Hg; *P* = 0.001) but not for criteria B (*P* = 0.347) or C (*P* = 0.670). The number of previous trabeculectomies decreased the risk for criterion C (RR = 0.73; *P* = 0.037) but not for criteria A (*P* = 0.115) or B (*P* = 0.371). The significant risk factors in Cox-proportional multivariable analysis were the fornix-based conjunctival flap [RR (95% CI) = 1.96 (1.21–3.21), *P* = 0.006] and a lower preoperative IOP [RR (95% CI) = 0.94 (0.90–0.98) per mm Hg, *P* = 0.001] for criterion A, the fornix-based conjunctival flap [RR (95% CI) = 1.60 (1.04–2.48), *P* = 0.032] for criteria B, and combined lens extraction [RR (95% CI) = 2.55 (1.03–6.40), *P* = 0.044] for criterion C (Table 5).

## Discussion

The aim of the present study was to determine whether limbus-based conjunctival incisions contributed to better surgical outcomes for trabeculectomy in OAG eyes that had prior ocular surgery. This prospective cohort study demonstrated that there were significantly higher failure rates in the fornix-based group than in the limbus-based group for criteria A and B. In addition, significantly higher cumulative probabilities of failure were observed in the fornix-based group for criteria A and B as well. Even after adjustment for other potential prognostic factors in the Cox proportional hazard regression models, the independent contribution of fornix-based conjunctival flap incision to the prediction of surgical failure was confirmed for both criteria A and B. Data indicated that limbus-based conjunctival incision was more beneficial for the surgical success of trabeculectomy in OAG eyes that had prior ocular surgery than fornix-based conjunctival incision.

There have been many prospective and retrospective studies about the surgical outcomes between limbus- versus fornix-based trabeculectomy. However, most of the studies have included smaller sample sizes than our present study. Prospective studies in 37 phakic POAG eyes by Shuster et al.^14^ and in 69 POAG eyes by Lemon et al.^15^ showed no significant difference of postoperative IOP between limbus- and fornix-based trabeculectomies. Other retrospective studies between limbus- and fornix-based trabeculectomies also resulted in no significant difference in surgical prognosis^16^,^17^, although lower postoperative IOPs with the limbus-based trabeculectomy procedures were found in 2 other studies^18^,^19^. These studies also included small sample sizes of 82 patients or fewer. Most studies that assigned limbus-based trabeculectomy to one eye and fornix-based trabeculectomy to the other eye for each patient resulted in no significant differences in surgical success between the 2 procedures^20^,^21^,^27^ with the exceptions of a better prognosis for the fornix-based trabeculectomy in the study by Brincker et al.^22^ and a lower postoperative IOP with limbus-based trabeculectomy in the study by Mandić et al.^23^ The sample sizes in these studies were 44 patients or fewer. A retrospective study of 215 glaucomatous eyes showed no significant differences in success between the 2 procedures^24^. Because these eyes had not undergone prior ocular surgeries, 97% of eyes in both procedure groups achieved a postoperative IOP of less than 20 mmHg. Another retrospective study in 797 eyes of 634 patients also found no significant difference between the 2 procedures^28^. This study failed to show that younger age or previous ocular surgery were associated with surgical failure, although many studies have presented these variables as risk factors^2^,^6^,^29^; this may be because the study included eyes with various glaucoma types and patients of both European and non-European descents. A retrospective study of 73 pseudophakic OAG patients including 67 patients of European descent showed that a fornix-based conjunctival flap incision was a risk factor for surgical failure^10^. This result implied that limbus-based trabeculectomy may be favorable for OAG eyes that had prior ocular surgery. Our present study is unique because it contained a large-scale cohort of patients with identical ethnicities which confirmed that limbus-based trabeculectomy was more effective for OAG eyes that had prior ocular surgery.

The reason that limbus-based trabeculectomy appears to be more effective for OAG eyes with prior ocular surgery remains unclear in the present study. A recent prospective study revealed that monocyte chemoattractant protein-1 and interleukin-8 in the aqueous humor were upregulated 1 to 2 years after phacoemulsification in cataract patients^7^. These inflammatory cytokines recruit leukocytes from the vessels. There have been many reports that state that limbus-based trabeculectomy created avascular blebs more frequently than fornix-based trabeculectomy^11^, which is consistent with our present data. The less vascularity after limbus-based trabeculectomy may restrict cytokine-mediated migration of leukocytes from the vessels to the bleb in pseudophakic eyes. Another possibility may be associated with the localized bleb after limbus-based trabeculectomy. Fornix-based trabeculectomy creates a more diffuse and extended bleb^12^. However, except for clear corneal phacoemulsification, eyes with prior ocular surgery have been associated with conjunctival scar formation, which can restrict the expansion of the bleb. This restricted conjunctival mobility may decrease the efficiency of the aqueous humor filtration in eyes treated with fornix-based trabeculectomy. Actually, among the patients recruited by CBIITS, the subgroup analysis for trabeculectomy in OAG eyes, which did not have the risk factors of previous ocular surgeries, i.e., <60 years of age or a preoperative IOP of <18 mm Hg, revealed that the probabilities of success at 3 years were 74.0% vs. 72.1% for criterion A (*P* = 0.535), 69.9% vs. 68.6% for criterion B (*P* = 0.631), and 52.0% vs. 53.2% for criterion C (*P* = 0.824) in the limbus- and fornix-based groups, respectively. Data were consistent with no significant difference between the 2 groups, similar to previous studies of glaucoma patients treated with primary trabeculectomy^14^,^15^,^16^,^17^,^20^,^21^,^24^,^27^. The third possibility may be due to the distance between the subconjunctival tissue and the scleral flap. Limbus-based trabeculectomy offers a higher bleb and is more frequently associated with cystic bleb formation than fornix-based trabeculectomy^11^,^12^. The conjunctiva in an eye with a surgical scar contains more fibroblasts^30^. When there is more distance between the subconjunctival tissue of the bleb and the sclera flap, there may be less wound healing at the scleral flap caused by the fibroblasts in the subconjunctival tissue. The fourth possibility is evaporation. Although the frequency of bleb leakage was not different between the two groups, it is possible that the thinner bleb wall more frequently exhibited micro-oozing or evaporation of aqueous humor through which might have resulted in better surgical outcome in the limbus-based group.

The other significant risk factors in the multivariable analysis of the present study were a lower preoperative IOP for criterion A and combined lens extraction for criterion C. The association of lower preoperative IOP with failure has also been reported by a previous study^28^. It would be more difficult to reduce IOP by a particular percentage for eyes with lower preoperative IOP. This risk factor may result from the specific definition of the criteria for surgical failure. Many studies have suggested that combined lens extraction was associated with surgical failure of trabeculectomy^31^,^32^,^33^,^34^. The present study showed no significant difference in the numbers of patients treated with combined lens extraction between the limbus- and fornix-based groups. For criterion C, no significant difference was found although a better surgical outcome was observed in the limbus-based group. A larger study may be needed to determine the true significance of this criterion.

One major limitation of the present study was that the patients were not randomly assigned to each group although there were no significant differences in the demographic data between the 2 groups. To confirm the present data with a randomized control trial, a large-scale sample size of more than 100 eyes in each arm would be required. Second, the surgical technique for trabeculectomy was not identical among the study centers or surgeons because this was established as a subgroup analysis for a prospective study of bleb-related infection. A randomized controlled trial would also be required to confirm the present results. Third, we could not identify the location and extent of the conjunctival surgical scarring after any previous surgeries. We were not able to determine the distance between the filtering bleb and the region of conjunctival scarring. However, the previous study evaluating the surgical outcomes in glaucomatous eyes with prior ocular surgery, which received either trabeculectomy or tube shunt surgery, did not determine conjunctival scarring either^8^. Fourth, the surgical outcomes of limbus-based trabeculectomy were not compared with those of tube shunt surgery. The probability of success at 3 years of the tube shunt group in the tube versus and trabeculectomy study was 84.9%^35^. Limbus-based trabeculectomy may still be less effective than tube shunt surgery for glaucoma eyes that had prior ocular surgery.

In conclusion, among patients with OAG who had undergone prior ocular surgery, limbus-based trabeculectomy offered a higher probability of surgical success for 2 of the 3 criteria, compared with fornix-based trabeculectomy. A trabeculectomy with MMC with a limbus-based conjunctival incision appears to be a surgical option for glaucomatous eyes at high risk of failure.

## Author Contributions

M.I. had full access to all of the data in the study and took responsibility for the integrity of the data and the accuracy of the data. Y.T. and M.I. designed study concept. S.Y., Y.T. and M.I. conducted the study. M.I. and CBIIT Study Group collected the data. S.Y., Y.T. and M.I. analyzed the data. S.Y., Y.T. and M.I. drafted the manuscript. All the author reviewed and approved the manuscript. CBIIT study group members were listed in Appendix 1 of the Reference No. 25.

## Figures and Tables

**Table 1:** Preoperative characteristics of the limbus- and fornix-based groups

	Limbus-Based Group (n = 106)	Fornix-Based Group (n = 89)	*P* value
Age (years), mean ± SD	69.0 ± 10.4	69.9 ± 12.2	0.60^a^
Sex, n (%)			0.54^b^
Man	63 (59.4%)	49 (55.1%)	
Woman	43 (40.6%)	40 (44.9%)	
Type of glaucoma, n (%)			0.29^b^
Primary open-angle glaucoma	79 (74.5%)	72 (80.9%)	
Exfoliation glaucoma	27 (25.5%)	17 (19.1%)	
Prior trabeculectomy, n (%)	49 (46.2%)	34 (38.2%)	0.26^b^
Prior lens extraction, n (%)	82 (77.4%)	72 (80.9%)	0.55^b^
Lens status, n (%)			0.57^c^
Phakic	24 (22.6%)	17 (19.1%)	
Pseudophakic	78 (73.6%)	66 (74.2%)	
Aphakic	4 (3.8%)	6 (6.7%)	
logMAR BCVA	0.41 ± 0.60	0.47 ± 0.64	0.46^a^
IOP (mm Hg), mean ± SD	24.6 ± 8.7	25.0 ± 9.5	0.74^a^
Glaucoma medications, mean ± SD	2.8 ± 1.2	3.1 ± 1.3	0.17^d^

BCVA = best-corrected visual acuity; IOP = intraocular pressure; logMAR = logarithm of minimum angle of resolution; SD = standard deviation.

^a^: t-test;

^b^: chi-square test;

^c^: exact permutation chi-square test;

^d^: Wilcoxon rank-sum test.

**Table 2:** Intraocular pressure and medical therapy at baseline and follow-up

	Limbus-based	Fornix-based	*P* value
**Baseline**			
** IOP (mm Hg)**	24.6 ± 8.7	25.0 ± 9.5	0.74^a^
** Number of medications**	2.8 ± 1.2	3.1 ± 1.3	0.17^b^
** n**	106	89	
**6M**			
** IOP (mm Hg)**	11.6 ± 4.7	13.6 ± 7.3	0.03^a^
** Number of medications**	0.4 ± 0.9	0.5 ± 0.9	0.56^b^
** n**	104	86	
**12M**			
** IOP (mm Hg)**	11.7 ± 4.3	12.6 ± 4.5	0.19^a^
** Number of medications**	0.7 ± 1.2	0.6 ± 1.0	0.44^b^
** n**	99	81	
**18M**			
** IOP (mm Hg)**	11.6 ± 4.5	12.7 ± 4.3	0.11^a^
** Number of medications**	0.7 ± 1.2	0.8 ± 1.1	0.44^b^
** n**	95	79	
**24M**			
** IOP (mm Hg)**	11.8 ± 4.1	13.6 ± 6.2	0.03^a^
** Number of medications**	0.8 ± 1.3	1.0 ± 1.2	0.15^b^
** n**	91	72	
**30M**			
** IOP (mm Hg)**	11.9 ± 4.5	13.6 ± 6.1	0.05^a^
** Number of medications**	0.9 ± 1.2	1.0 ± 1.2	0.49^b^
** n**	90	67	
**36M**			
** IOP (mm Hg)**	12.5 ± 5.9	14.1 ± 6.4	0.12^a^
** Number of medications**	1.0 ± 1.4	1.0 ± 1.2	0.48^b^
** n**	86	66	

Data shown in mean ± standard deviation

IOP = intraocular pressure.

^a^; t-test,

^b^; Wilcoxon rank-sum test.

**Table 3:** Postoperative complications of the limbus- and fornix-based groups

	Limbus-Based Group (n = 106)	Fornix-Based Group (n = 89)	*P* value
Cataract progression	5 (4.7%)	1 (1.1%)	0.13
Avascular bleb	37 (34.9%)	14 (15.7%)	0.002
Bleb leak	5 (4.7%)	8 (9.0%)	0.23
Bleb infection	1 (0.9%)	1 (1.1%)	0.90
Loss of light perception	0 (0.0%)	1 (1.1%)	0.21
Re-operation	5 (4.7%)	5 (5.6%)	0.78
Deterioration of BCVA			
BCVA ≧ 0.2	49 (46.2%)	37 (41.6%)	0.51
BCVA ≧ 0.3	34 (32.1%)	26 (29.2%)	0.67
logMAR BCVA ≧0.3	35 (33.0%)	31 (34.8%)	0.79

BCVA = best-corrected visual acuity; logMAR = logarithm of minimum angle of resolution.

**Table 4:** Relative risk analyzed by univariate Cox proportional hazards regression models

	Criteria					
	A		B		C	
	RR (95% CI)	*P*	RR (95% CI)	*P*	RR (95% CI)	*P*
**Age per year**	0.99 (0.98–1.02)	0.79	1.00 (0.98–1.01)	0.69	1.00 (0.98–1.01)	0.63
**Sex (female/male)**	1.44 (0.89–2.29)	0.13	1.40 (0.92–2.14)	0.12	1.27 (0.87–1.84)	0.21
**Type of glaucoma(XFG/POAG)**	0.83 (0.43–1.46)	0.53	0.84 (0.47–1.41)	0.52	0.93 (0.58–1.45)	0.76
**The number of prior trabeculectomy per each**	0.85 (0.58–1.21)	0.37	0.76 (0.53–1.07)	0.12	0.73 (0.52–0.98)	0.04
**Lens status**						
**(pseudophakia/phakia)**	0.81 (0.48–1.43)	0.45	1.01 (0.62–1.74)	0.96	1.24 (0.78–2.05)	0.37
**(aphakia/phakia)**	0.67 (0.16–1.97)	0.50	1.25 (0.42–3.12)	0.66	1.29 (0.47–3.00)	0.60
**Preoperative logMAR per 1.0**	0.84 (0.55–1.22)	0.37	1.02 (0.72–1.40)	0.91	1.00 (0.74–1.32)	0.99
**IOP per mm Hg**	0.95 (0.91–0.98)	<0.01	0.99 (0.96–1.01)	0.35	1.00 (0.98–1.03)	0.67
**The number of preoperative glaucoma medication per each**	1.01 (0.83–1.23)	0.94	1.06 (0.88–1.26)	0.55	1.08 (0.92–1.26)	0.36
**Combined surgery (Combined/alone)**	1.96 (0.95–3.66)	0.07	1.45 (0.70–2.66)	0.30	1.42 (0.72–2.54)	0.29
**Conjunctival incision (Fornix-based/limbus-based)**	2.00 (1.25–3.23)	<0.01	1.71 (1.12–2.62)	0.01	1.33 (0.92–1.93)	0.13

IOP = intraocular pressure; logMAR = logarithm of minimum angle of resolution; POAG = primary open-angle glaucoma; XFG = exfoliation glaucoma.

**Table 5:** Relative risk analyzed by multivariate Cox proportional hazards regression models

	Criteria					
	A		B		C	
	RR (95% CI)	*P*	RR (95% CI)	*P*	RR (95% CI)	*P*
**Age per year**	0.99 (0.97–1.01)	0.42	0.99 (0.97–1.01)	0.40	0.99 (0.97–1.01)	0.22
**Sex (female/male)**	1.34 (0.81–2.21)	0.25	1.31 (0.84–2.06)	0.23	1.19 (0.80–1.76)	0.39
**Type of glaucoma(XFG/POAG)**	1.31 (0.44–0.65)	0.44	1.04 (0.56–1.86)	0.89	0.97 (0.58–1.60)	0.92
**The number of prior trabeculectomy per each**	0.78 (0.47–1.23)	0.29	0.73 (0.46–1.09)	0.12	0.74 (0.51–1.05)	0.09
**Lens status**						
**(pseudophakia/phakia)**	1.17 (0.48–3.08)	0.73	1.16 (0.51–2.84)	0.72	1.59 (0.76–3.61)	0.22
**(aphakia/phakia)**	1.30 (0.26–5.11)	0.72	1.53 (0.42–5.04)	0.50	1.54 (0.48–4.64)	0.46
**Preoperative logMAR per 1.0**	1.02 (0.66–1.51)	0.94	1.08 (0.75–1.50)	0.68	1.02 (0.74–1.37)	0.89
**IOP per mm Hg**	0.94 (0.90–0.98)	<0.01	0.99 (0.96–1.01)	0.28	1.00 (0.98–1.02)	0.93
**The number of preoperative glaucoma medication per each**	0.98 (0.80–1.20)	0.83	1.01 (0.84–1.22)	0.89	1.05 (0.89-–1.25)	0.54
**Combined surgery (Combined/alone)**	2.29 (0.85–6.34)	0.10	1.89 (0.73–5.00)	0.19	2.55 (1.03–6.40)	0.04
**Conjunctival incision (Fornix-based/limbus-based)**	1.96 (1.21–3.21)	<0.01	1.60 (1.04–2.48)	0.03	1.23 (0.84–1.80)	0.29

IOP = intraocular pressure; logMAR = logarithm of minimum angle of resolution; POAG = primary open-angle glaucoma; XFG = exfoliation glaucoma.
